# Supplementary material for: Passing the Message: Representation Transfer in Modular Balanced Networks
Source: Front Comput Neurosci. 2019 Dec 5;13:79. doi: 10.3389/fncom.2019.00079 (PMC6915101; doi:10.3389/fncom.2019.00079)
Supplement: Supplementary file 1 [file Data_Sheet_1.pdf]

Supplementary Material: Passing the message: representation transfer in modular balanced networks

Barna Zajzon[1,2*], Sepehr Mahmoudian[4,5], Abigail Morrison[1,3], Renato Duarte[1]

1 *Institute of Neuroscience and Medicine (INM-6), Institute for Advanced Simulation (IAS-6) and JARA Institute Brain Structure-Function Relationships (JBI-1 / INM-10), Jülich Research Centre, Jülich, Germany*
2 *Department of Psychiatry, Psychotherapy and Psychosomatics, RWTH Aachen University, Germany*
3 *Institute of Cognitive Neuroscience, Faculty of Psychology, Ruhr-University Bochum, Germany*
4 *Department of Data-driven Analysis of Biological Networks, Campus Institute for Dynamics of Biological Networks, Georg August University Göttingen, Germany*
5 *MEG Unit, Brain Imaging Center, Goethe University, Frankfurt, Germany*

Correspondence*:
Barna Zajzon
b.zajzon@fz-juelich.de

1 SUPPLEMENTARY FIGURES

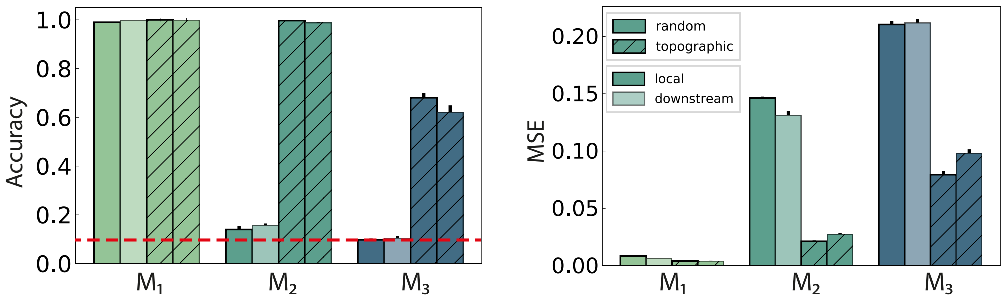

Figure S1. Classification accuracy and mean squared error (MSE) computed using ten stimuli from the second input stream, S'. There are no significant differences between local and downstream integration.

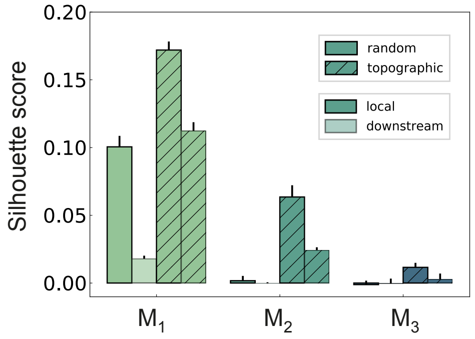

Figure S2. Silhouette score quantifying cluster separability in the XOR task. Scores are calculated in the space spanned by the first ten PCs, using the low-pass filtered spike trains as the main state variable in the analysis.

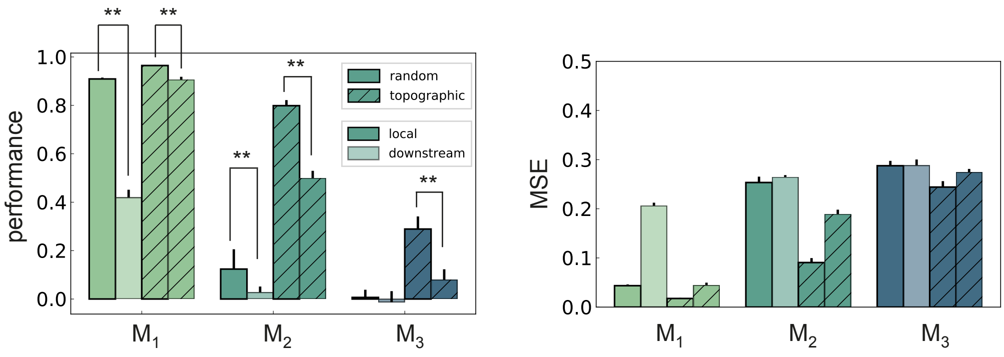

Figure S3. Performance on the XOR task (point-biserial correlation coefficient), computed using the low-pass filtered spike trains as the main state variable. The differences in performance are statistically significant, with local integration proving to be consistently more beneficial. These results are in agreement with the values computed using the membrane potentials as state variables.

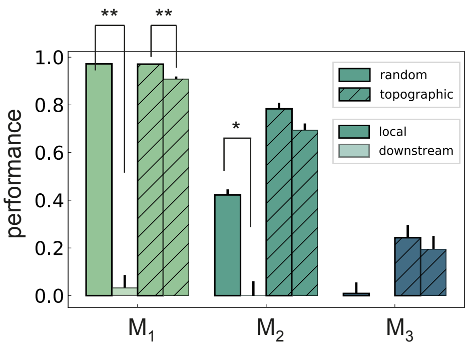

Figure S4. Performance on the XOR task for networks with non-scaled feed-forward projections between $M_0 \rightarrow M_1$ and $M_0' \rightarrow M_1$ in the downstream integration scenario (see Figure 6B in the main text). The denser connectivity does not significantly alter the relative differences between local and downstream integration.

2 SUPPLEMENTARY TABLES

**A: Model Summary**	
**Populations**	Multiple modules, each one composed of 1 excitatory and 1 inhibitory sub-population
**Topology**	None
**Connectivity**	Sparse, random recurrent connectivity with random or topographically structured feed-forward projections
**Neuron Model**	Leaky integrate-and-fire, fixed voltage threshold, fixed absolute refractory time, no adaptation
**Synapse Model**	Conductance-based, exponential
**Plasticity**	None
**Input**	Stochastic background spikes and inhomogeneous Poisson spikes onto 10%E and 10% I neurons
**Measurements**	Spiking activity, membrane potentials

**B: Populations**		
**Name**	**Elements**	**Size**
E_i, E'_0	`iaf_cond_exp`	8000
I_i, I'_0	`iaf_cond_exp`	2000

**C: Neuron Models**	
**Name**	Leaky integrate-and-fire neuron (`iaf_cond_exp`)
**Subthreshold Dynamics**	if $(t > t^* + \tau_{\mathrm{ref}})$<br>$C_{\mathrm{m}} \frac{dV_i}{dt} = \mathrm{g}_{\mathrm{leak}}(V_{\mathrm{rest}} - V_i(t)) + I_i^{\mathrm{E}}(t) + I_i^{\mathrm{I}}(t) + I_i^{\mathrm{x}}(t)$<br>else<br>$V(t) = V_{\mathrm{reset}}$
**Synaptic Transmission**	$I_{\mathrm{ij}}^{\mathrm{syn}}(t) = g_{\mathrm{ij}}^{\mathrm{syn}}(V_{\mathrm{syn}} - V_i(t))$
**Spiking**	If $V(t-) < V_{\mathrm{th}}$ OR $V(t+) \geq V_{\mathrm{th}}$<br>1. set $t^* = t$ 2. emit spike with time stamp t^*

**D: Synapse Models**	
**Synaptic Conductance**	$\frac{dg_{\mathrm{ij}}(t)}{dt} = -\frac{g_{\mathrm{ij}}(t)}{\tau_\beta} + \bar{g}^\beta \sum_{t_j} \delta(t - t_j - d)$

**E: Input**		
**Type**	**Target**	**Description**
poisson_generator	E_0, I_0	Total rate $\nu_{\mathrm{X}} \cdot \mathrm{K}_{\mathrm{X}}$
poisson_generator	E_i, I_i for $i > 0$	Total rate $0.25 \cdot \nu_{\mathrm{X}} \cdot \mathrm{K}_{\mathrm{X}}$
inhomogeneous_poisson_generator	$\mathrm{E}_0^{(k)}$, $\mathrm{I}_0^{(k)}$ for $S_k \in S$<br>$\mathrm{E}_0^{\prime(j)}$, $\mathrm{I}_0^{\prime(j)}$ for $S'_j \in S'$	Inhomogeneous Poisson process with rate ν_{stim}, changing every 200 ms

**F: Measurements**
Spiking activity, membrane potentials

Table S1. Tabular description of network model after Nordlie et al. (2009).

A: Populations		
**Name**	**Value**	**Description**
N^{E}	8000	Excitatory population size in each module
N^{I}	2000	Excitatory population size in each module
**B: Connectivity**		
**Name**	**Value**	**Description**
d	$1.5\,\mathrm{ms}$	Synaptic transmission delay
$\overline{g}_{\mathrm{E}}$	1 nS	Excitatory synaptic conductance
$\overline{g}_{\mathrm{I}}$	$\gamma\overline{g}_{\mathrm{E}}$ nS	Inhibitory synaptic conductance
γ	16	Scaling factor for the inhibitory synapses
ϵ	0.1	Baseline connection probability
p_{x}	ϵ	Connection probability for background noise input in M_0
	0.25ϵ	Scaled connection probability for background input in $M_i, i > 0$
p_{ff}	0.75ϵ	Feed-forward connection probability within topographic maps
**B: Neuron Model**		
**Name**	**Value**	**Description**
C_{m}	250 pF	Membrane capacitance
E_L	$-70\,\mathrm{mV}$	Resting membrane potential
τ_{m}	15 ms	Membrane time constant
V_{th}	$-50\,\mathrm{mV}$	Membrane potential threshold for action-potential firing
V_{reset}	$-60\,\mathrm{mV}$	Reset potential
τ_{ref}	2 ms	Absolute refractory period
g_{L}	$16.7\,\mathrm{nS}$	Leak conductance
**C: Synapse Model**		
τ_{E}	$5\,\mathrm{ms}$	Synaptic decay time constant for excitatory synapses
τ_{I}	$10\,\mathrm{ms}$	Synaptic decay time constant for inhibitory synapses
V_{E}	$0\,\mathrm{mV}$	Excitatory reversal potential
V_{I}	$-80\,\mathrm{mV}$	Inhibitory reversal potential

Table S2. Summary of all the model parameters.

3 SUPPLEMENTARY DATA

The code package provided as a supplement (Supplementary File 1) implements project-specific functionality to NMSAT (Duarte et al., 2017), which is a tailor-made Python package that provides a generic set of tools to build, simulate and analyse neuronal microcircuit models with any degree of complexity, as exemplified in this study. It provides a high-level wrapper for PyNEST (used as the core simulation engine). To use the provided software:

1. Setup - After ensuring that all dependencies are satisfied, NMSAT[1] version 0.2 needs to be downloaded and setup, as explained in the provided documentation[2].
2. Project code - The code package for this project should then be extracted onto the `projects/` folder. The provided code has the following structure:

[1] `https://github.com/rcfduarte/nmsat`

[2] `https://rcfduarte.github.io/nmsat/`

```
state_transfer/
├── parameters/
│   └── preset/
├── computations/
└── read_data/
```

where `read_data` contains the scripts necessary to read, analyse and plot the data. The main simulations are run using combinations of parameters files with the corresponding computation function (see Table S2 for a description of the experiments provided and the standard use case in the code documentation for instructions).

3. Running a simulation - Specific experiments can be run from scratch using the provided code. Modify the specific parameters as desired (paying particular attention to the system specificities) and execute the experiment:

```
$ python main.py -f {parameters_file} -c {computation} --extra {computation_parameters}
```

The code package is also available online at the following Open Science Framework repository: https://osf.io/nywc2/.

Experiment	Parameters file (.py)	Computation
Stimulus classification in random sequential hierarchies (Fig.2 A,B; Fig.4)	random_sequential_class	stimulus_processing
Stimulus classification in topographic sequential hierarchies (Fig.2 A,B; Fig.4)	topographic_sequential_class	
Modulating stimulus amplitude in random networks (Fig.2 E)	random_modulate_amplitude	
Modulating connection density within topographic maps (Fig.2 F)	topographic_modulate_density	
Influence of direct connections $M_0 \Rightarrow M_1$ (Fig.2 C,D)	random_direct_connections	remove_direct_connections
Population activity statistic in the noise-driven scenario (Fig.3)	stats_noise	char_population_activity
Population activity statistic in the random, stimulus-driven scenario (Fig.3)	stats_random	
Population activity statistic in the random, stimulus-driven scenario (Fig.3)	stats_topographic	
Stimulus sensitivity and memory capacity in random networks (Fig.5)	random_sequential_memory	stimulus_processing_memory
Stimulus sensitivity and memory capacity in topographic networks (Fig.5)	topographic_sequential_memory	
Multi-stream classification and XOR in random networks with local integration (Fig.6 C, and Fig7. A,B,C)	random_integrate_local	stimulus_integrate
Multi-stream classification and XOR in random networks with downstream integration (Fig.6 C, and Fig7. A,B,E)	random_integrate_downstream	
Multi-stream classification and XOR in topographic networks with local integration (Fig.6 C, and Fig7. A,B,D)	topographic_integrate_local	
Multi-stream classification and XOR in topographic networks with downstream integration (Fig.6 C, and Fig7. A,B,F)	topographic_integrate_downstream	
XOR with mixed input in random networks (Fig.8 C,D,E)	random_mixing_downstream	
XOR with mixed connectivity in random networks (Fig.8 F,G,H)		

Table S3. Summary of all the numerical experiments that can be run using the provided source code.

REFERENCES

Duarte, R., Zajzon, B., and Morrison, A. (2017). Neural Microcircuit Simulation And Analysis Toolkit doi:10.5281/ZENODO.582645

Nordlie, E., Gewaltig, M.-O., and Plesser, H. E. (2009). Towards reproducible descriptions of neuronal network models. *PLoS computational biology* 5, e1000456
